# C-752-03. Performance and Bias of AI-Assisted Burn Depth Assessment Compared with Clinician Accuracy

**DOI:** 10.1093/jbcr/irag033.016

**Published:** 2026-04-06

**Authors:** Moon Usman, Katerina Andreadis, Roselle E Crombie

**Affiliations:** Riverside Community Hospital, Riverside, CA; NYU Langone, NY, New York; Connecticut Burn Center/Yale New Haven Health, Westport, CT

## Abstract

**Introduction:**

Artificial intelligence (AI) systems are increasingly investigated for burn wound depth assessment, with reported accuracies that exceed traditional clinical evaluation. However, the ethical and clinical safety of AI-assisted assessment remain underexplored, particularly regarding performance bias across skin tones, liability, and regulatory oversight. This study aimed to evaluate the accuracy and equity of AI models in burn assessment and to identify considerations for safe clinical deployment.

**Methods:**

A structured literature review (2010–2025) of peer-reviewed studies and FDA regulatory documents was performed using PubMed, Scopus, and FDA repositories. Inclusion criteria were AI or machine learning applications for burn imaging with reported accuracy metrics or subgroup analyses. Seven clinical studies with image-based AI classifiers were pooled using fixed- and random-effects models with binomial variance approximations. Additional optical modality studies and reviews were included for context on dataset representation and regulatory frameworks.

**Results:**

Human clinicians correctly identified partial-thickness burn depth in about two-thirds of cases, with an average accuracy of 67% (95% CI: 63–70%). In contrast, pooled results from seven AI studies (n = 3050 images) showed significantly higher performance: Fixed-effects model: 85.0% accuracy (95% CI: 83.8–86.2%). Random-effects model: 84.9% accuracy (95% CI: 83.6–86.1%). Despite these gains, important limitations emerged. Fewer than 10% of dataset images represented darker skin tones (Fitzpatrick V–VI). In subgroup analyses, AI systems made 22% more errors on darker skin and showed ~15% lower sensitivity on yellow-hued skin tones (*p*<.05). From a regulatory perspective, the FDA’s 2025 draft guidance on AI/ML medical devices requires ongoing monitoring and subgroup reporting, while the 2024 final guidance on Predetermined Change Control Plans (PCCPs) establishes conditions for updating AI models to reduce bias and clarify liability.

**Conclusions:**

AI-assisted burn assessment demonstrates superior pooled accuracy compared with clinician visual assessment but introduces equity concerns due to dataset imbalance and subgroup disparities. Future applications include burn triage in emergency departments, telemedicine consultation for non-specialists, longitudinal wound monitoring to reduce unnecessary visits, and integration into reconstructive planning. However, regulatory frameworks only partially mitigate liability but require fairness testing and transparent governance.

**Applicability of Research to Practice:**

There are a multitude of uses for technology in plastic and burn surgery that includes triage, more accurate wound assessments despite experience and possible reduction in length of stay for patients needing operative intervention.

**Funding for the Study:**

N/A.

